# Tissue resident memory T cells are enriched and dysfunctional in effusion of patients with malignant tumor

**DOI:** 10.7150/jca.83615

**Published:** 2023-05-05

**Authors:** Xueying Mao, Yue Chen, Xiulian Lu, Shuiping Jin, Piao Jiang, Zhangfeng Deng, Xiaoyun Zhu, Qichun Cai, Changyou Wu, Shuangpeng Kang

**Affiliations:** 1 Clinical Research Center of Clifford Hospital, Guangzhou, P.R. China;; 2 Cancer Center of Clifford Hospital, Guangzhou, P.R. China;; 3 Obstetrics of Clifford Hospital, Guangzhou, P.R.China;; 4 Academician Workstation, Hunan Key Laboratory of the Research and Development of Novel Pharmaceutical Preparations, Changsha Medical University, Changsha, P.R. China.

**Keywords:** Malignant effusion, Tissue resident memory T cells, PD-1, Cancer.

## Abstract

**Purpose** Most malignant effusion is secondary to metastases to the pleura or peritoneum and portend poor oncological outcomes. Malignant effusion has different tumor microenvironment from primary tumor, containing a variety of cytokines and immune cells and directly contacting with tumor cells. However, the characteristic of CD4^+^ T cells and CD8^+^ T cells in malignant effusion remains unclear.

**Methods** Malignant effusion including peritoneal ascites and pleural fluid from thirty-five patients with malignant tumor were collected and compared with matched blood. A detailed characterization of CD4^+^ T cells and CD8^+^ T cells in malignant effusion were conducted using flow cytometry and multiple cytokines assay.

**Results** The concentration of IL-6 in malignant effusion was significantly higher than in blood. A substantial portion of T cells in malignant effusion were CD69^+^ and/ or CD103^+^ Trm cells. Most CD4^+^T and CD8^+^T cells in malignant effusion were exhausted T cells which expressed lower levels of cytokines, cytotoxic molecules and markedly higher levels of inhibitory receptor PD-1 compared with in blood.

**Conclusion** Our study is the first to identify the presence of Trm cells in malignant effusion and will lay the foundation for future research on anti-tumor immunity of Trm cells in malignant effusion.

## Introduction

Malignant effusion is common in patients with cancer. Most malignant effusion are secondary to metastases to the pleura or peritoneum and the presence of malignant effusion often indicates advanced disease and poor survival 1, 2. Malignant effusion has different tumor microenvironment from primary tumor, containing a variety of cytokines and immune cells and directly contacting with tumor cells. It has advantages such as easy acquisition and often used as a mirror to reflect the tumor microenvironment 3-5. Malignant effusion has high levels of immune suppressive cytokines such as VEGF, IL-10, and TGF-β, which are directly or indirectly related to angiogenesis and cancer progression 3, 6-8. Some studies reported that pro-inflammatory cytokines IL-6, TNF-α and IFN-γ were also elevated in malignant pleural effusion, whereas others did not 9. Certain types of immune cells were identified as independent prognostic factors and used to immune monitoring because of its accessibility. Proportion of CD8^+^ T lymphocyte with memory marker (CD45RO) and activation marker (HLA-DR), CD3 T lymphocyte with PD-1, and number of FoxP3^+^ Tregs were identified as independent prognostic factors in metastatic gastric cancer 3. However, immune cells in malignant effusion can be continuously exchanged between effusion and tumor tissue, and/or between blood 10. This dynamic immunoenvironment leads to divergent results in different studies.

Conventionally, memory cells could be parsed into two subsets, which were labeled central memory T cells (Tcm) and effector memory T cells (Tem) cells, according to their homing characteristics and effector functions. In recent years, another distinct group of memory T cells has attracted more and more attention and has been named tissue resident memory T cells (Trm) cells based on their tissue resident properties. Trm cells seed in peripheral tissues without recirculating and provide a rapid and robust local response to re-infection at body surfaces. Long-lived Trm cells are observed in different tissues and play a crucial role in pathogen clearance, autoimmune diseases, tumorigenesis 11-13. Cavity-resident macrophages which expressed high levels of Tim-4 were associated with reduced numbers of CD8^+^ T cells with tumor-reactive features in pleural effusion and peritoneal ascites from patients with metastatic cancer 14. Activated CD4^+^, CD8^+^ and regulatory T-cells accumulated in malignant ascites from ovarian carcinoma patients 5. However, no literature on the characteristic of tissue resident memory T cells in malignant effusion has been published.

Here, we conducted a detailed characterization of CD4^+^ T cells and CD8^+^ T cells in malignant effusion including peritoneal ascites and pleural fluid from thirty-five patients with malignant tumor and compared with results from matched blood. We found that the concentration of IL-6 in malignant effusion was significantly higher than in blood, suggesting that these cytokines were produced locally in the pleural or peritoneal cavity. Moreover, we showed that a substantial portion of T cells in malignant effusion were CD69^+^ and/ or CD103^+^ Trm cells and dysfunctional which expressed lower levels of cytokines and cytotoxic molecules and markedly higher levels of PD-1 compared with blood.

## Materials and methods

### Subjects

A total of 35 patients with malignant tumor (17 females and 18 males, 29-80 years old) were recruited from Clifford Hospital of Guangzhou, China. Matched blood and malignant effusion including peritoneal ascites and pleural fluid were collected from patients with malignant tumor. Patients with malignant effusion were diagnosed by detection of malignant cells in the effusion and/or biopsied specimens. Patients who had been diagnosed with HIV, HBV, HCV or who had a history of autoimmune diseases were excluded from the study. Written informed consents were obtained from all patients. Ethics approval for the present study was obtained from the ethics committee of Clifford Hospital.

### Cell Isolation

Venous blood and malignant effusion including ascites and pleural fluid were collected and centrifuged at 2000 rpm for 10 min. The supernatant of malignant effusion and plasma were collected for multiplex cytokine assay. The pelleted cells were resuspended in PBS and loaded onto Ficoll-Hypaque (Tianjin HaoYang Biological Manufacture, China) and centrifuged at speed 2200 rpm/min for 20 min. The mononuclear cells on upper layer were collected and washed with PBS twice. The cells were counted and adjusted to a density of 2 × 10^6^ cells/mL in complete RPMI-1640 medium for next study.

### Flow Cytometry and MAbs

Cells were washed and suspended in 100 μL of PBS containing 0.1% BSA and 0.05% sodium azide. For surface staining, cells were incubated with the respective mAbs at 4°C in the dark for 30 min. For the detection of intracellular cytokines, cells were fixed with 4% paraformaldehyde and permeabilized in PBS buffer containing 0.1% saponin (Sigma-Aldrich, USA), 0.1%BSA and 0.05% sodium azide for at least 2 h or overnight at 4°C and stained with conjugated mAbs for intracellular cytokines. Flow cytometry data were acquired with FACS Canto II (BD Bioscience, USA) and analyzed with FlowJo software (Tree Star, USA). The following mAbs were used for cell surface or intracellular staining: FITC labeled anti-CD3, anti-CD45RA, anti-Perforin, APC-labeled anti-CD69, anti-TNF-a, anti-PD-1, PE-labeled anti-CD103, anti- IFN-γ, anti-GranzymeB, Percp labeled anti-CD3, PE-cy7 labeled anti-CD4, Apc-cy7 labeled anti-CD8 and isotype matched control antibodies were all purchased from BD Bioscience PharMingen (San Jose, CA, USA).

### Cytokine assay

Multiple cytokines (IL-2/IL-4/IL-6/IL-10/TNF-α/IFN-γ) levels of malignant effusion and plasma were measured simultaneously using the IL-2/IL-4/IL-6/IL-10/ TNF-α/IFN-γ Test Assay (#8931028; Aligent, Hangzhou, China) according to the manufacturer′s instructions. Serial dilution of standard and samples (volume 25 μL) incubated with the magnetic beads 2h at room temperature according to the kit instructions.The samples were acquired using FACS Canto II equipment. Cytokine concentrations were determined by LEGEND plex Software.

### Statistical analysis

Data were presented as the mean±standard error of mean (SEM). Statistical significance was analyzed by Mann-Whitney test using Prism 6 (GraphPad, San Diego, CA, USA). Significant p-values were indicated in figures for the following ranges: NS, no significance; *, P < 0.05; **, P <0.01; ***, P < 0.001; ****, P < 0.0001.

## Results

### The levels of IL-6 and IL-10 in malignant effusion were significantly higher than that in blood

**Figure 1:**
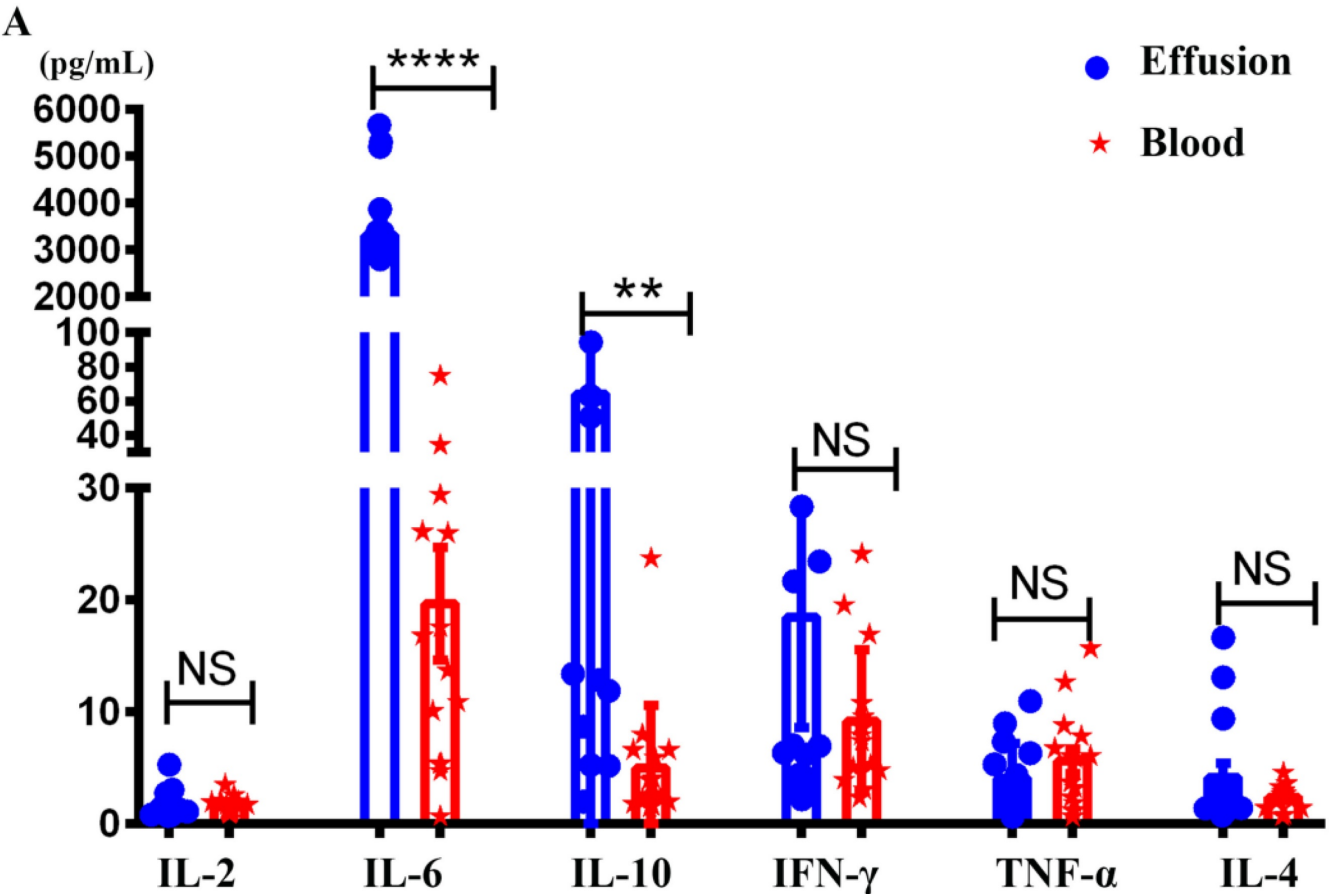
** The concentrations of different cytokines in malignant effusion and blood.** The supernatant of malignant effusion and blood were prepared and the concentrations of IL-2/IL-4/IL-6/IL-10/TNF-α/IFN-γ were determined by flow cytometry** (A)**. Statistical results from 15 independent experiments were shown as mean±SEM, and the statistical significance was determined with the Mann-Whitney U test. NS, no significance, **<0.01, ****P < 0.0001.

The study consisted of a variety of primary cancer types and patient ages, with gastric cancers being the most frequent (Table 1). Malignant effusions were of volumes between 100-1000 mL and contained between 0.4×10^6^ and 5.1 × 10^8^ total cells with an average of 4.5 × 10^7^ ± 1.76 × 10^7^ cells per effusion. The levels of IL-2, IL-4, IL-6, IL-10, TNF-α and IFN-γ in the supernatant of malignant effusion and blood were assessed by multiple cytokines detection kit. The results showed that the concentration of IL-6 in malignant effusion (3311 ± 367.8) was significantly higher than that in the blood (19.70 ± 5.05). The concentration of IL-10 in malignant effusion (64.28 ± 17.15) was higher than that in the blood (5.02 ± 1.45). However, there was no difference between malignant effusion and blood in the concentration of IL-2, IFN-γ, TNF-α and IL-4 (Figure 1A).

### The absolute numbers of different subsets of T cells in malignant effusion were significantly lower than in blood, but there was no difference in the proportion

**Figure 2:**
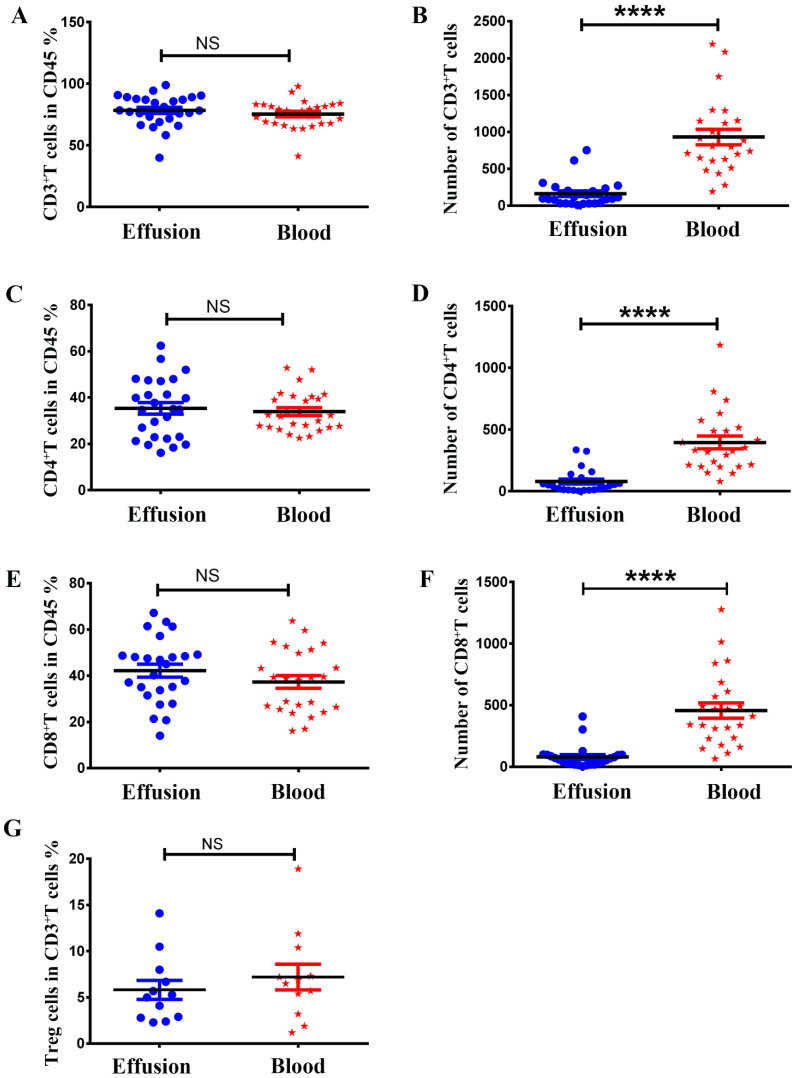
** The proportion and absolute numbers of different subsets of T cells in malignant effusion and blood.** The proportion and absolute numbers of T cells (CD45^+^CD3^+^) **(A, B)**, CD4^+^T cells (CD45^+^CD3^+^CD4^+^) **(C, D)**, CD8^+^T cells (CD45^+^CD3^+^CD8^+^) **(E, F)**, Treg cells (CD45^+^CD3^+^CD4^+^CD25^+^CD127^-^) **(G)** in malignant effusion and blood were detected by flow cytometry. Statistical results were shown as mean±SEM. The statistical significance was determined with the Mann-Whitney U test. NS, no significance, ****P < 0.0001.

As shown in Figure 2, there was no difference between malignant effusion and blood in the proportion of T cells (Figure 2A), CD4^+^T cells (Figure 2C), CD8^+^T cells (Figure 2E), Treg cells (Figure 2G). However, the absolute numbers of T cells (Figure 2B), CD4^+^T cells (Figure 2D), CD8^+^T cells (Figure 2F) per microliter in malignant effusion were significantly lower than that in blood.

### Trm cells accounted for a sizeable portion of T cells in malignant effusion

**Figure 3:**
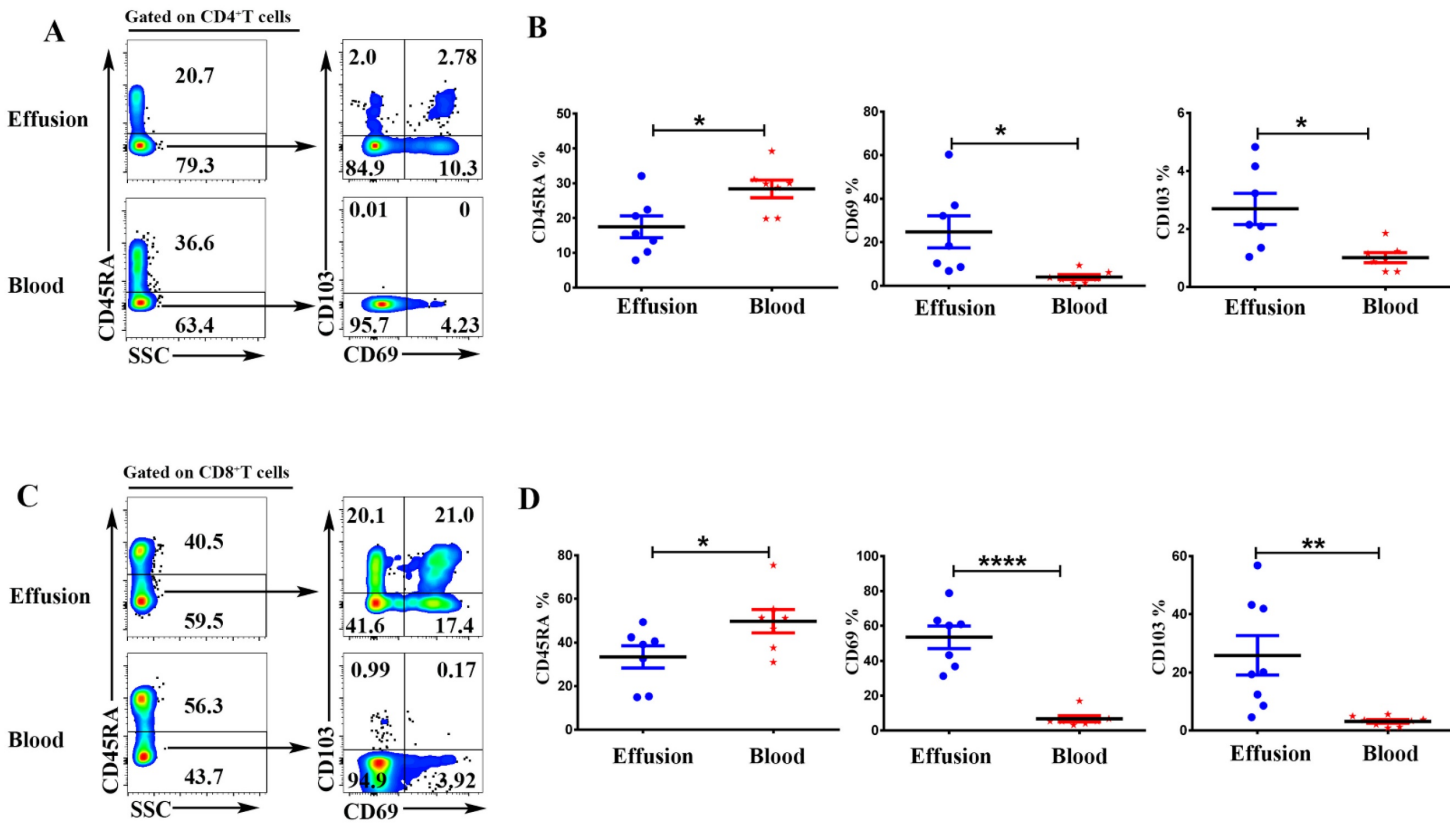
** The expression of CD45RA, CD69, CD103 on CD4^+^T and CD8^+^T cells in malignant effusion and blood.** The mononuclear cells from malignant effusion and blood were prepared and stained with anti-CD45, CD3, CD4, CD8, CD45RA, CD69, and CD103, and assessed by FACS. Gated on CD3^+^CD4^+^T cells **(A, B)** and CD3^+^CD8^+^T cells **(C, D)**, the representative graphs for the expression of CD45RA, CD69, and CD103 were shown. Statistical results from 7 independent experiments were shown as mean ± SEM. The statistical significance was determined with the Mann-Whitney U test. *P<0.05, **<0.01, ****P < 0.0001.

To compare the expression of memory and tissue resident markers, the mononuclear cells from malignant effusion and blood were analyzed by FACS after cell surface staining. As shown in Figure 3, Gated on CD3^+^CD4^+^ cells or CD3^+^CD8^+^ cells, CD4^+^T and CD8^+^T cells in malignant effusion were found to be predominantly CD45RA^-^, thereby exhibiting a memory cell phenotype. CD4^+^T cells from malignant effusion expressed higher levels of CD69 and CD103 than that from blood (Figure 3A, 3B). Moreover, the majority of CD8^+^T cells in malignant effusion were CD69^+^CD103^+^, CD69^+^CD103^-^, or CD69^-^CD103^+^ tissue resident memory T cells, and expressed significantly higher levels of CD69 and CD103 than that from blood (Figure 3C, 3D).

### CD4^+^T and CD8^+^T cells in malignant effusion expressed lower levels of IFN-γ and TNF-α than that in blood

**Figure 4:**
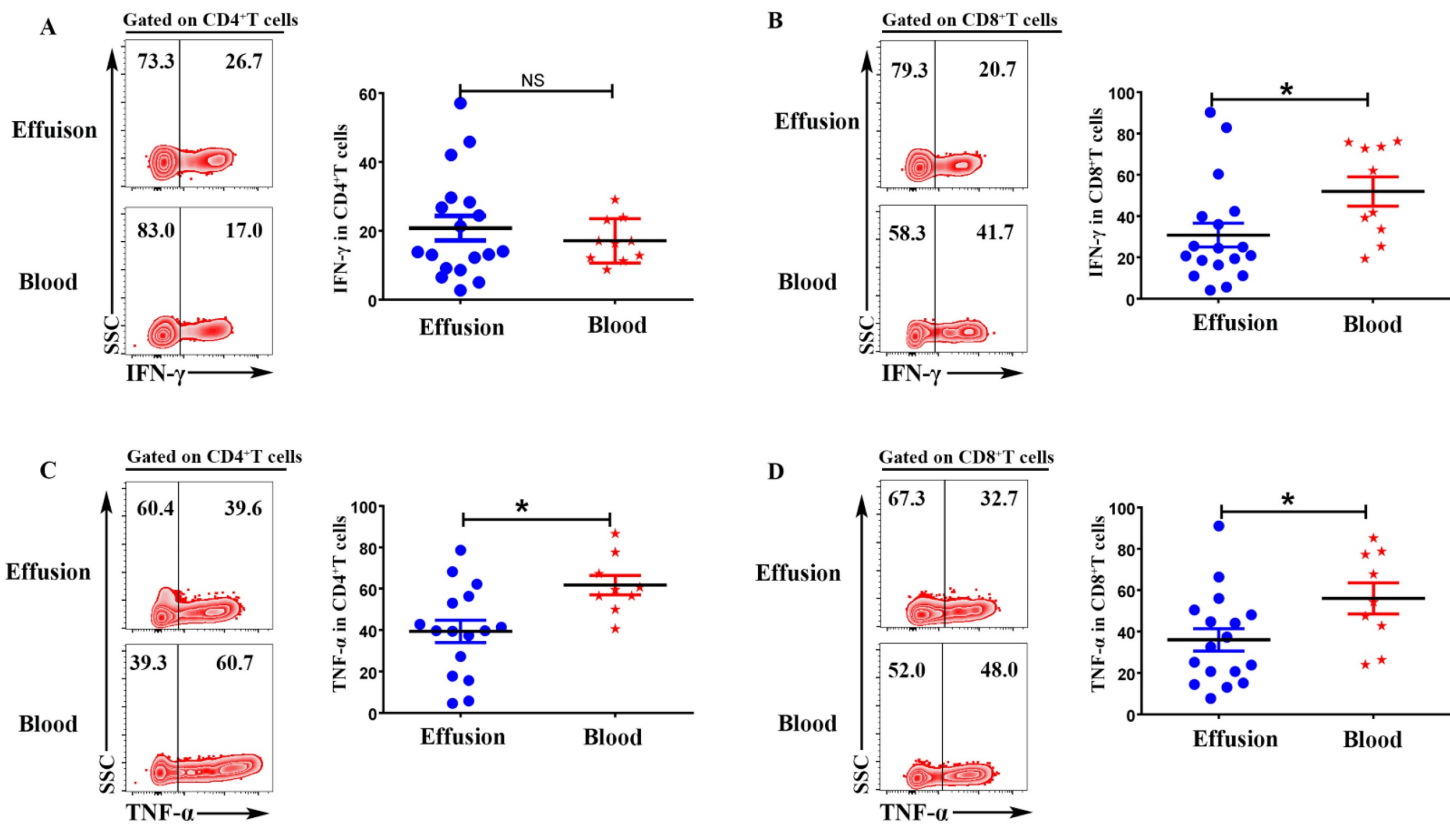
** CD4^+^T and CD8^+^T cells in malignant effusion expressed lower levels of IFN-γ and TNF-α than that in blood.** The mononuclear cells from malignant effusion and blood were stimulated with or without PMA plus ionomycin in the presence of BFA for 6 h and analyzed by FACS. Gated on CD3^+^CD4^+^T **(A, C)** and CD3^+^CD8^+^T cells **(B, D)**, The representative graph and statistical results for the expression IFN-γ and TNF-α were shown. Statistical results were shown as mean±SEM. The statistical significance was determined with the Mann-Whitney U test. NS, no significance; *P < 0.05.

To evaluate the expression of IFN-γ and TNF-α on CD4^+^T and CD8^+^T cells, the mononuclear cells from malignant effusion and blood were stimulated with or without PMA plus ionomycin for 6 h in the presence of BFA. The results from FACS data demonstrated that there was no difference on the expression of IFN-γ in CD4^+^T cells (Figure 4 A). CD8^+^T cells from malignant effusion (30.78 ± 5.79) expressed lower levels of IFN-γ than that in blood (51.93 ± 7.09) (P < 0.05) (Figure 4 B). In addition, both CD4^+^T and CD8^+^T cells in malignant effusion expressed lower levels of TNF-α than that in blood (Figure 4C, D).

### CD4^+^T and CD8^+^T cells in malignant effusion expressed lower levels of Granzyme B and Perforin than that in blood

**Figure 5:**
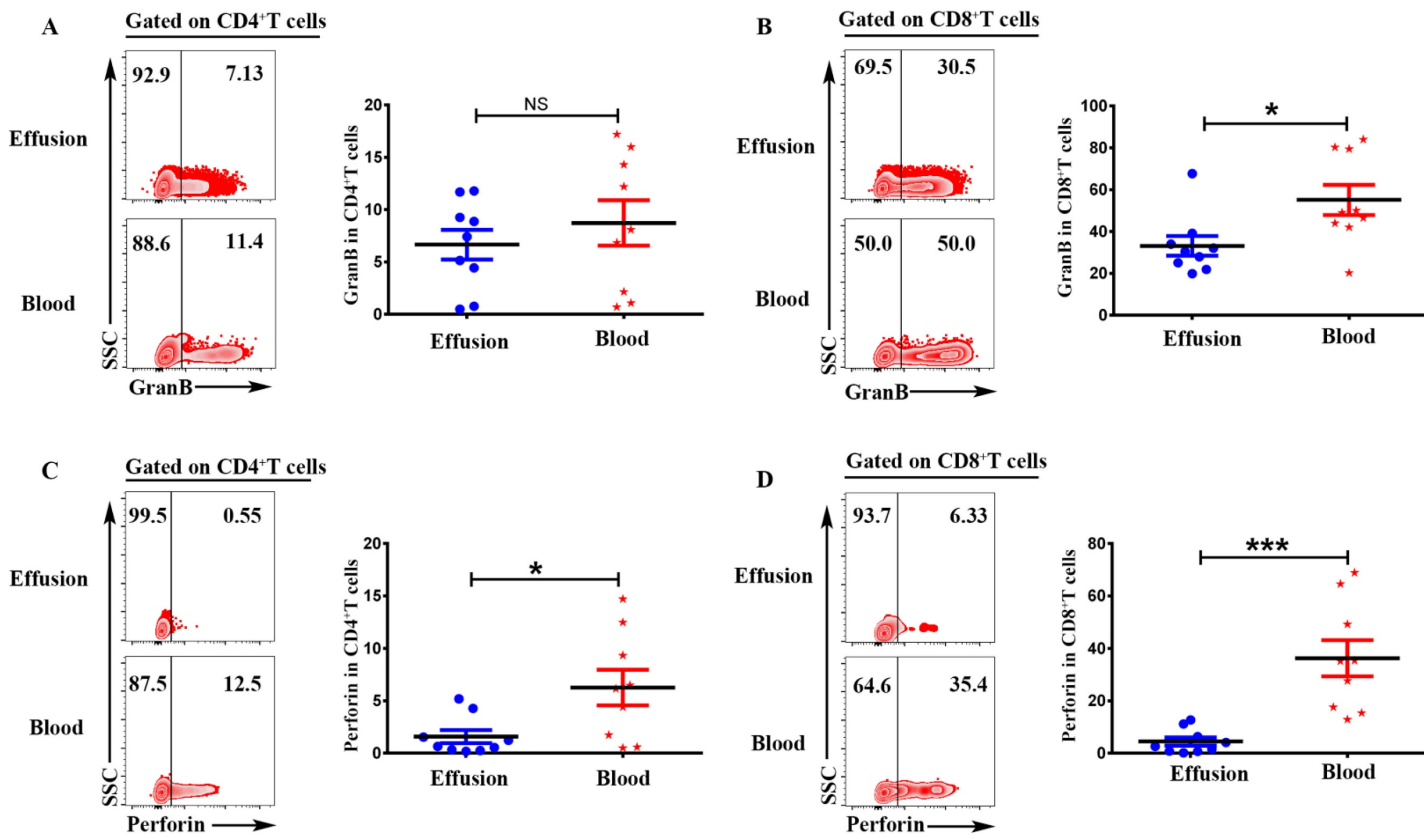
**CD4^+^T and CD8^+^T cells in malignant effusion expressed lower levels of Granzyme B and Perforin than that in blood.** The mononuclear cells from malignant effusion and blood were prepared and stained with anti-CD45, CD3, CD4, CD8, Granzyme B and Perforin, and assessed by FACS. Gated on CD3^+^CD4^+^T** (A, C)** and CD3^+^CD8^+^T cells** (B, D)**, the representative graph and statistical results for the expression Granzyme B and Perforin were shown. Statistical results were shown as mean±SEM. The statistical significance was determined with the Mann-Whitney U test. NS, no significance; *P < 0.05; ***P < 0.001.

To investigate the cytotoxic activity of CD4^+^T and CD8^+^T cells in malignant effusion and blood, the expression of Granzyme B and Perforin were assessed by FACS. The results showed that there was no difference on the expression of Granzyme B in CD4^+^T cells from malignant effusion and blood (Figure 5A). CD8^+^T cells from malignant effusion (33.14 ± 4.76) expressed lower levels of Granzyme B than that in blood (55.08 ± 7.17) (P < 0.05) (Figure 5B). CD4^+^T cells from malignant effusion (1.58 ± 0.62) expressed lower levels of Perforin than that in blood (6.27 ± 1.70) (P < 0.05) (Figure 5C). Furthermore, CD8^+^T cells from malignant effusion (4.48 ± 1.56) expressed extremely lower levels of Perforin than that in blood (36.33 ± 6.92) (P < 0.001). Those results indicated that T cells in malignant effusion had a lower cytotoxic activity than T cells in blood.

### CD4^+^T and CD8^+^T cells in malignant effusion expressed significantly higher levels of PD-1 than that in blood

**Figure 6:**
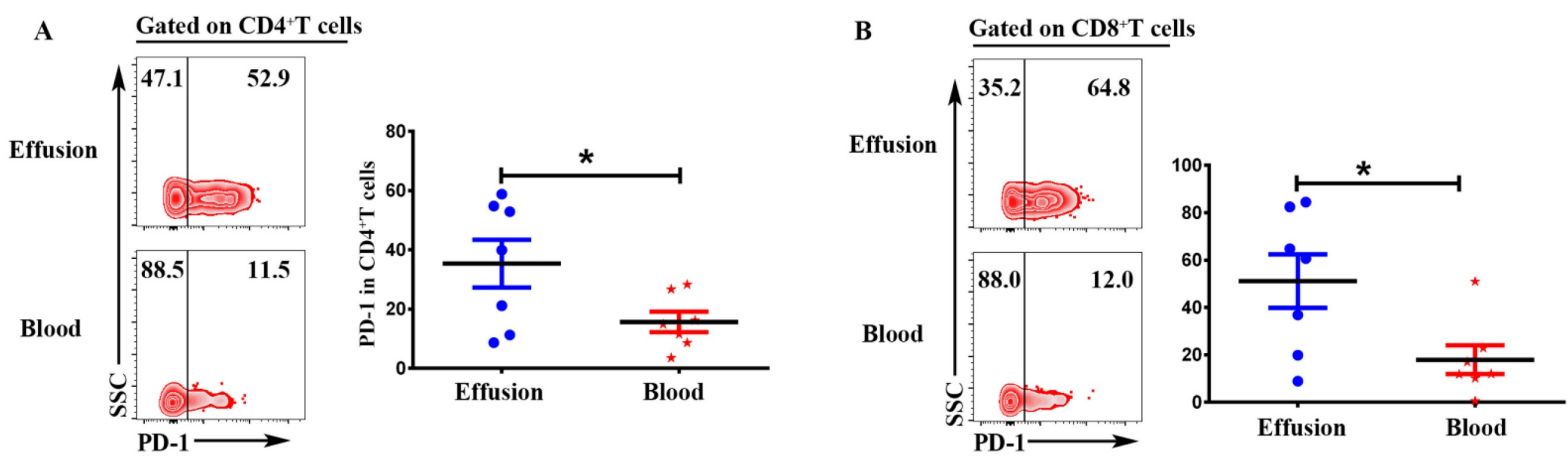
**CD4^+^T and CD8^+^T cells in malignant effusion expressed significantly higher levels of PD-1 than that in blood.** The mononuclear cells from malignant effusion and blood were prepared and stained with anti-CD45, CD3, CD4, CD8, and PD-1, and assessed by FACS. Gated on CD3^+^CD4^+^T **(A)** and CD3^+^CD8^+^T cells **(B)**, The representative graph and statistical results for the expression PD-1were shown. Statistical results were shown as mean ± SEM. The statistical significance was determined with the Mann-Whitney U test. *P < 0.05.

Expression of inhibitory receptor PD-1 in T cells from malignant effusion was assessed and compared to paired peripheral blood T cells. The FACS data showed that CD4^+^T in malignant effusion (35.37 ± 8.08) expressed significantly higher levels of PD-1 than that in blood (15.68 ± 3.44) (P < 0.05) (Figure 6A). In addition, PD-1 was up-regulated markedly on CD8^+^T cells from malignant effusion (51.17 ± 11.27) compared to from blood (17.93 ± 6.08) (P < 0.05) (Figure 6B). In the end, the comparisons of immunological characteristic between malignant effusion and blood T cells were listed in Table 2.

## Discussion

Most of metastatic cancer are accompanied by malignant effusion including peritoneal ascites and pleural fluid and portend poor oncological outcomes. Malignant effusion consists of cancer cells, mesothelial cells, and vasious immune cells including abundant T cells, natural killer (NK) cells, B cells, macrophages, and neutrophils. Each of these cells produces various chemokines and cytokines that lead to formation of tumor microenvironment in malignant effusion 4. The malignant effusion including pleural and peritoneal cavities represent immunosuppressive environments which enables the tumor cells to escape from immune surveillance 15, 16.

Pro-inflammatory cytokine IL-6 play a central role in chronic inflammatory including cancers. The IL-6/JAK/STAT signaling pathway is aberrantly hyperactivated in many types of cancer. Blockade of the IL-6 signalling pathway has become a target for the therapy of diverse cancers. IL-6 was released by several cell populations in malignant effusion, including cancer cells, macrophages, mesothelial cells 17-20. In this study, the concentration of IL-6 in malignant effusion reached up to 3311pg/mL, whereas much lower (19.7 pg/mL) in blood, suggesting IL-6 was produced locally in the pleural or peritoneal cavity rather than exuding form blood. By contrast, pro-inflammatory Th1 cytokines including IL-2, TNF-α, IFN-γ had not noticeably altered, consistently with the study in malignant pleural effusion of non-small cell lung cancer 20. Immune modulatory cytokine IL-10 has paradoxical effects on different types of immune response and is considered as a potential switcher of immunity. It can inhibit the production of pro-inflammatory cytokines and activation of T cells. It also has been reported to exert anti-tumor effects through promoting the proliferation and cytotoxicity of CD8^+^T cells 21-23. Our study showed that the concentration of IL-10 in malignant effusion (64.28 pg/mL) was slightly higher than that in the blood (5.02 pg/mL).

Trm cells are derived from precursors that entered tissues during the effector phase of immune responses and remained long-term within this compartment. In addition to protecting against local infections, Trm cells have been reported to suppress tumor growth and strongly correlate with favorable prognosis in cancer patients 11, 12, 24, 25. The identification of Trm cells in peripheral tissues delineated two key markers that are expressed by the majority of these cells: CD69 and CD103 (αE integrin), both of which are gradually up-regulated during Trm cells development. CD69 can bind to S1PR1 and trap early activated T cells in secondary lymphoid organs until they are fully primed. Similarly, upregulation of CD69 both play functional roles in the development and retention of Trm cells. CD103 binds to E-cadherin expressed on epithelial cells to anchor Trm cells to epithelial tissues 11, 26, 27. In this study, we found that a substantial portion of T cells in malignant effusion were Trm cells highly expressing CD69 and/or CD103, especially CD8^+^T cells. Different from T cells in blood, 24.7 % of CD4^+^T cells and 53.4% of CD8^+^T cells expressed CD69, and 25.8% of CD8^+^T cells expressed CD103. These results indicated that most tumor‐infiltrating T cells in malignant effusion were resident in pleural and peritoneal cavities without recirculating.

In chronic infections and cancer, persistent antigen and/or inflammatory signals cause the deterioration of T cell function: a state called 'exhaustion'. Exhausted T cells lose robust effector functions and express multiple inhibitory receptors. Different from T cell anergy, revitalization of exhausted T cells can reverse dysfunction and reinvigorate immunity. T cell exhaustion is a progressive process. Firstly, functions such as IL-2 production and cytokine polyfunctionality, as well as high proliferative capacity are lost. Secondly, the deficiency in the production of IFN-γ, TNF and chemokines, as well as in degranulation is followed. In addition, T cell exhaustion is also accompanied by a progressive increase in the amount and diversity of inhibitory receptors such as PD-1, Tim-3, et al. And finally, if the the antigen persists in the long-term, the antigen-specific T cells can be lost 28, 29. Our results indicated that CD4^+^T cells and CD8^+^T cells in malignant effusion were dysfunctional which expressed lower levels of IFN-γ, TNF-α, Granzyme B and Perforin than in blood. Higher and sustained expression of inhibitory receptors is a hallmark of exhausted T cells. The programmed death 1 (PD-1)/programmed death ligand 1 (PD-L1) and/or PD-L2 axis is the more extensively studied inhibitory signalling pathway and correlated with early progression and shorter survival in cancer 30. CD4^+^T and CD8^+^T cells in malignant effusion expressed significantly higher levels of PD-1 than that in blood. Together, these data indicated that most CD4^+^T and CD8^+^T cells in malignant effusion were exhausted T cells and lost robust effector function.

In summary, our study showed that a substantial portion of T cells in malignant effusion were Trm cells without recirculating which highly expressed CD69 and/or CD103. Most CD4^+^T and CD8^+^T cells in malignant effusion were exhausted T cells which expressed lower levels of cytokines, cytotoxic molecules and markedly higher levels of inhibitory receptor PD-1 compared with in blood. To our best knowledge, our study is the first to identify the presence and proportion of Trm cells in malignant effusion. The function of these Trm cells in malignant effusion is needed to be further studied.

## Figures and Tables

**Table 1:** The tumor types and clinical characteristics of subjects.

Characteristics	Patients with Malignant effusion (n=35)
Age years (mean ± SD)	53.77±10.20
Male/Female	18/17
Primary tumor	
Gastric	7 (20.0%)
Pancreatic	4 (11.4%)
Breast	6 (17.1%)
Lung	5 (14.3%)
Ovarian	3 (8.57%)
Colorectum	3 (8.57%)
Nasopharynx	2 (5.71%)
Liver	2 (5.71%)
Other	3 (8.57%)

**Table 2:** The immunological characteristic of T cells in malignant effusion and blood.

Name	Maliganat effusion	Blood	p value
Cytokines (pg/mL)			
IL-2	1.867 ± 0.308	1.629 ± 0.184	0.5145
IL-6	3311 ± 367.8	19.70 ± 5.045	< 0.0001
IL-10	64.28 ± 17.15	5.018 ± 1.446	0.0018
IFN-γ	18.47 ± 9.842	9.214 ± 1.635	0.3615
TNF-α	4.109 ± 0.793	5.609 ± 1.125	0.2852
IL-4	4.151 ± 1.258	2.395 ± 0.268	0.1832
Memory (%)			
CD45RA in CD4	17.47 ± 3.130	28.36 ± 2.558	0.0195
CD45RA in CD8	33.46 ± 5.087	49.69 ± 5.355	0.0484
Residency (%)			
CD69 in CD4	24.76 ± 7.395	3.939 ± 1.090	0.0165
CD69 in CD8	53.43 ± 6.359	6.686 ± 1.762	< 0.0001
CD103 in CD4	2.693 ± 0.539	1.014 ± 0.173	0.0118
CD103 in CD8	25.86 ± 6.714	3.136 ± 0.591	0.0046
Cytokines (%)			
IFN-γ in CD4	20.73 ± 3.580	17.10 ± 2.032	0.4805
IFN-γ in CD8	30.78 ± 5.791	51.93 ± 7.088	0.0332
TNF-α in CD4	39.35 ± 5.357	61.66 ± 4.644	0.0103
TNF-α in CD4	35.99 ± 5.355	55.96 ± 7.548	0.0397
Cytotoxicity (%)			
Granzyme B in CD4	6.463 ± 1.390	8.640 ± 2.151	0.4079
Granzyme B in CD8	33.14 ± 4.760	55.08 ± 7.174	0.0215
Perforin in CD4	1.578 ± 0.621	6.269 ± 1.704	0.0199
Perforin in CD8	4.480 ± 1.555	36.33 ± 6.915	0.0004
Inhibitory receptor (%)			
PD-1 in CD4	35.37 ± 8.084	15.68 ± 3.438	0.0446
PD-1 in CD8	51.17 ± 11.27	17.93 ± 6.079	0.0234

## Abbreviations

Tcm: Central memory T cells
Tem: Effector memory T cells
Trm: Tissue resident memory T cells
